# Cognitive map formation through haptic and visual exploration of tactile city-like maps

**DOI:** 10.1038/s41598-021-94778-1

**Published:** 2021-07-27

**Authors:** Loes Ottink, Marit Hoogendonk, Christian F. Doeller, Thea M. Van der Geest, Richard J. A. Van Wezel

**Affiliations:** 1 Donders Institute for Brain, Cognition and Behaviour, Radboud University, Nijmegen, The Netherlands; 2 Psychology Department, Max Planck Institute for Human Cognitive and Brain Sciences, Leipzig, Germany; 3 Kavli Insitute for Systems Neuroscience, NTNU, Trondheim, Norway; 4 Lectorate Media Design, HAN University of Applied Sciences, Arnhem, The Netherlands; 5 Techmed Centre, Biomedical Signals and System, University of Twente, Enschede, The Netherlands

**Keywords:** Learning and memory, Spatial memory, Cognitive neuroscience, Perception

## Abstract

In this study, we compared cognitive map formation of small-scale models of city-like environments presented in visual or tactile/haptic modalities. Previous research often addresses only a limited amount of cognitive map aspects. We wanted to combine several of these aspects to elucidate a more complete view. Therefore, we assessed different types of spatial information, and consider egocentric as well as allocentric perspectives. Furthermore, we compared haptic map learning with visual map learning. In total 18 sighted participants (9 in a haptic condition, 9 visuo-haptic) learned three tactile maps of city-like environments. The maps differed in complexity, and had five marked locations associated with unique items. Participants estimated distances between item pairs, rebuilt the map, recalled locations, and navigated two routes, after learning each map. All participants overall performed well on the spatial tasks. Interestingly, only on the complex maps, participants performed worse in the haptic condition than the visuo-haptic, suggesting no distinct advantage of vision on the simple map. These results support ideas of modality-independent representations of space. Although it is less clear on the more complex maps, our findings indicate that participants using only haptic or a combination of haptic and visual information both form a quite accurate cognitive map of a simple tactile city-like map.

## Introduction

Spatial navigation and wayfinding in familiar and unfamiliar environments are important but complex abilities. To successfully find our way, we have to remember and mentally represent the layout of our environment correctly. Studies show that the brain can form a neural representation of space, as a cognitive map of an environment^1,2^, which can support navigation and wayfinding. A cognitive map as considered here, is a mental representation of a small-scale model of an environment. It contains at least an allocentric (environment-centered, map-like) representation, and not only egocentric (body-centered, route-like) representations^1,3^. Such a cognitive map includes various types of spatial information, such as distances between locations^4–6^. It allows to employ allocentric (environmental-centered, map-like) as well as egocentric (body-centered, route-like) perspectives^1,3^. It therefore supports navigation of specific routes, but also to infer information that is not explicitly learned, such as detours and Euclidean distances. In the current study, we want to contribute to knowledge about the formation of cognitive maps. We aim to assess cognitive map formation through haptic map exploration, and how this compares to map acquisition by visual map learning, with increasing map complexity.

Many studies that assess cognitive map formation and the representation of spatial information, use visual input. Cognitive map formation has been suggested since at least the early 1900s^7^, mentioned as imaginary maps. It has been studied in rodents^2,8–10^, and was later extended to humans^1,5,6,11–17^. Less is known about these processes when an environment is represented through a non-visual sensory modality. This would be relevant when vision is less or not available, for instance for people with a visual impairment. In the current study, we focused on the tactile/haptic modality. The ability to form a cognitive map of a haptically presented environment has been suggested in earlier research, considering people with a visual impairment as well as sighted people^18–23^. Furthermore, there is a large body of literature that shows the effectiveness of tactile maps to support blind people in wayfinding and orientation^18,21,24–31^. There are several methods to assess the formation of mental spatial representations^32^. Research on cognitive map formation from haptic input investigated several spatial aspects, such as recall or reproduction of particular routes^18,24,25,33^ or wayfinding by directly holding and using a tactile map^18,34^. Furthermore, some studies use configurations of tactile objects and tested the formed mental representations by asking questions about the layout or recalling object locations^35,36^. Reproduction of map configurations has also been used to study cognitive maps^22,27,37,38^, as well as estimation of distances between learned haptic locations^12,13,35,39,40^. Most studies, however, address only a limited amount of such cognitive map aspects. In the current study, we aim to assess a more comprehensive view on cognitive maps by combining several types of spatial information and relations^1^. Hereby, we considered allocentric as well as an egocentric perspectives^1,3^.

We furthermore want to assess how haptic map learning compares to visual map learning, especially with increasing map complexity. This may give more insight into multimodal aspects of map learning, and allows to investigate whether vision is advantageous or whether information from both modalities lead to similar representations. Vision is the modality that for most people provides most detailed information and is the main sensory input used for the formation of cognitive maps in sighted persons^33,41,42^. Furthermore, haptic map learning is thought to be sequential, and a representation is gradually built up, while visually, information is processed as a whole^29,43^. On the contrary, there is also a growing body of evidence indicating a modality-independent coding of space in the brain^44–51^. This literature points to the integration of multiple input modalities into one amodal spatial representation, rather than the formation of a separate representation for each modality. For instance, highly similar patterns of spatial representations from the haptic and visual modalities supports this hypothesis, and mental spatial representations have been found to be nearly independent of modality^49,52^. Furthermore, the same brain regions are activated during spatial tasks involving different modalities or types of spatial information^45,51^.

In short, the main aim of this study is therefore to investigate whether a comprehensive cognitive map can be formed through navigating and learning a tactile map of a city-like environment and how this compares to visual map learning with increasing map complexity. To this end, participants explore three tactile city maps of differing complexity. On the maps, five locations are marked and associated with a unique item. Our participants explored the tactile maps during a short learning period, probably sufficient to learn with vision. We wanted to assess whether an equally accurate cognitive map could be formed with only haptic information in this relatively short learning period. After exploring each map, participants estimate Euclidean and path distances between item pairs, rebuild the map, recall the locations of the items, and navigate two routes. Hereby, we will investigate whether people accurately integrate information like street layout, item locations, and relationships between locations, in a cognitive map.

## Methods

### Participants

A group of 18 sighted participants (10 male, 8 female) was recruited to participate in this study. Participants were assigned to a vision-restricted, haptic (H) group (n = 9, 5 males; age range 20–27, mean age 21) or a sighted, visuo-haptic (VH) group (n = 9, 5 males; range 21–25, mean age 22). This sample size was calculated using behavioral data of previous studies (under review) and pilot experiments (calculated using G*Power; with effect size d = 1.44, α = 0.05 and power 0.9). All participants were university students, right-handed, had no cognitive or hearing problems, and had (corrected to) normal vision. Ethical approval for the study was given by the local ethical committee of the Radboud University, Nijmegen, The Netherlands. The experiments were performed in accordance with guidelines and regulations relevant for research involving human participants. All participants gave written informed consent before the start of the experiment.

Participants in the H group were visually occluded by a curtain such that all task-relevant information was taken away, while participants in the VH group could see everything during all tasks. A curtain was used for restriction of vision, but participants could still look around the experiment room. This was done because not having any visual input would be an unusual and possibly uncomfortable situation for sighted persons^53,54^.

### Tactile maps

**Figure 1:**
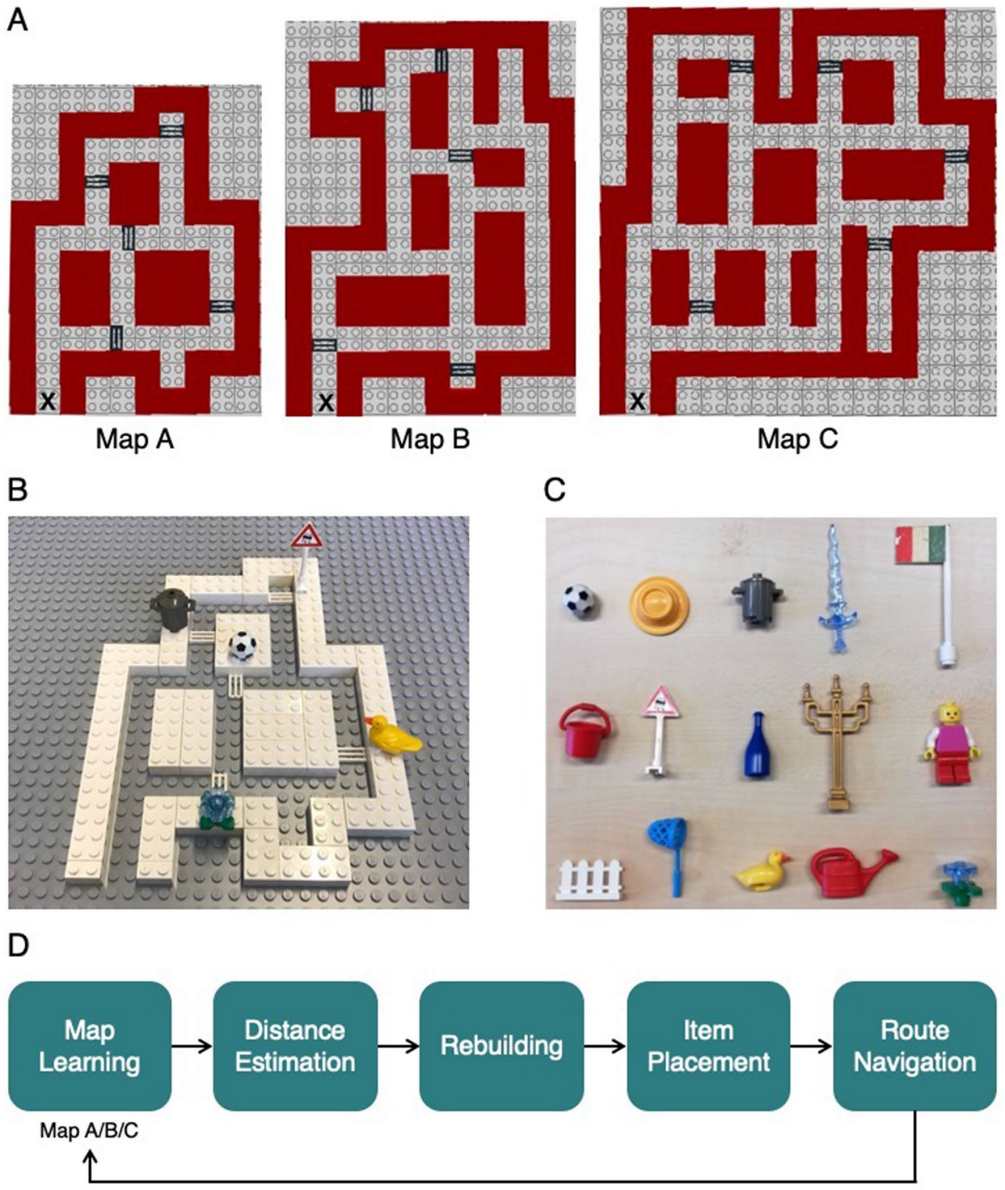
The tactile maps. (**A**) Top view of the three tactile maps and the items. Map A, B and C have 7, 11, and 15 intersections respectively. The five item locations are shown on each map (striped rectangles). The start locations are indicated with an ‘x’. (**B**) An example of map A including placement of the items. (**C**) The 15 used items. (**D**) Order of the five tasks that are performed for all three maps.

Three tactile maps were used in the experiment. The maps differed in complexity, which was defined by the number of intersections (7, 11, and 15 intersections; Fig. 1A). Each map had five marked locations, associated to a unique item. Each map had a fixed starting position, which was the same for all participants (Fig. 1A).

The maps were built using LEGO bricks, and were approximately 25 × 25 cm. The streets were lower than the surroundings, and had a width of 1.6 cm. The surroundings had a height of 1 regular LEGO building brick (1 cm). The item locations were recognizable by a ribbed texture (the location markings), and the items were placed right next to the corresponding location marking, on the surrounding (Fig. 1B). The locations were chosen such that some pairs had similar Euclidean and path distance, and some had not.

The items associated to the locations were easily identifiable tactile items. They were different on each map, so there were 15 items in total (Fig. 1C). They were randomized across the total of 15 locations for each participant. All items were presented to the participants before the start of the experiment, to make sure all participants could identify the tactile items. Participants in the H group identified the items solely haptically, and participants in the VH group could see as well as feel the items.

### Experimental session

The experiment consisted of three parts, corresponding to the three tactile maps. Participants performed tasks for each of the three maps, in counterbalanced order, such that each map was explored as the first map by an equal number of participants.

The task for the participants of the experiment was to explore and learn the layout of each map, and to learn the locations of the items. After learning each map, the participants performed four tasks to measure their knowledge and cognitive map formation: They first had to estimate relative distances between each item pair, then rebuild the map using LEGO bricks, place the items on the right locations, and finally navigate the shortest route between two items (Fig. 1D).

All tasks were explained before the start of the experiment, so participants knew what was going to happen before each part. Participants in the H group were visually occluded by a curtain during all tasks, while the VH group was never vision-restricted. Both groups always received the same instructions. At the end of the experiment, participants were asked about their learning strategies and whether they feel they performed better on the last map because of experience. They were also asked about wayfinding in unfamiliar environments in daily life: whether they plan the route beforehand and try to form a mental map, whether they use a navigational app while navigating in the environment, or a mixed strategy.

### Map learning

#### Task

Participants learned each map by free navigation and exploration using their right index finger, starting at the start location (Fig. 1A). We thereby monitored the participants, to make sure they actually only used their right index finger. The map was introduced as the street map of a city. Participants were instructed to navigate as if they were actually on the location on the map of their index finger, and as if they walked through the city in that way. They were asked to learn the locations and the associated items, which they could identify using their right hand. The maximum learning time was 10 min. Participants could stop earlier if they felt they knew the map.

### Distance estimation task

#### Task

In the distance estimation task, participants had to estimate relative Euclidean and path distances between each item pair. Here, Euclidean distance was the distance of a straight line between two items, and path distance was the distance of the shortest route between the items. The maps were taken away from the participants before the start of this task.

Participants had to indicate their estimations on a ruler. The most left position on the ruler would mean that the items are on the same location, and the rightmost position would mean that the items were located the furthest apart possible on the corresponding map. The other distances were scaled to this. For each estimation, the experimenter handed the two items of the pair to the participant to identify. Then, the Euclidean distance between the item pair was asked first, followed by the path distance. This was repeated for all pairs. Participants in both groups indicated their estimation by pointing at a position on the ruler using their right index finger. The experimenter made sure that the VH participants could not see the numbering on the ruler.

#### Analysis

We correlated the estimated distances with the correct distances, for both the Euclidean and path distance. We furthermore performed an error analysis to look at over- or underestimation. All estimated and correct distances were first normalized as a proportion of the maximum distance possible. Then, the error was calculated for each item pair, for both Euclidean and path distance, for each participant. These errors were corrected for the maximum error possible on the map, since this differs for each item pair. The mean error was computed across item pairs for each participant, for Euclidean as well as path distance. All analyses were performed in MATLAB (MATLAB and Statistics Toolbox Release 2012b, The MathWorks, Inc., Natick, Massachusetts, United States).

### Rebuilding task

#### Task

In the rebuilding task, participants had to rebuild the map with LEGO bricks as accurately and completely as possible. Thereby, they had to build the paths on an empty building plate. No time limitations were given, and they could use an unrestricted number of bricks. Both groups performed this task in their respective visual conditions, so the participants in the H group were visually occluded by the curtain while the VH participants were not.

#### Analysis

Before analysing the rebuilt maps, we created standardized images of those maps. Scores were given to each map using the Gardony Map Drawing Analyzer (GMDA; Software for quantitative analysis of sketch maps, version 1, https://www.aarongardony.com/tools/map-drawing-analyzer)^55^.This software compares drawn maps to an original map based on the organization of chosen landmarks, and gives accuracy scores based on different kinds of calculations.

For this analysis, we chose each turn and intersection in the original map and the rebuilt maps to be a landmark (see Figure A.1 in the supplemental material for an example). We used two types of accuracy scores, canonical organization and distance accuracy scores. Canonical organization scores are calculated by first determining North/South and East/West relationships for each landmark pair on the original map, and then for the rebuilt map. Of these relationships, the proportion of correct ones on the rebuilt map is the canonical organization score^55^. An example of this analysis is given in Appendix A (Figure A.2, Supplementary Table 1).

**Figure 2:**
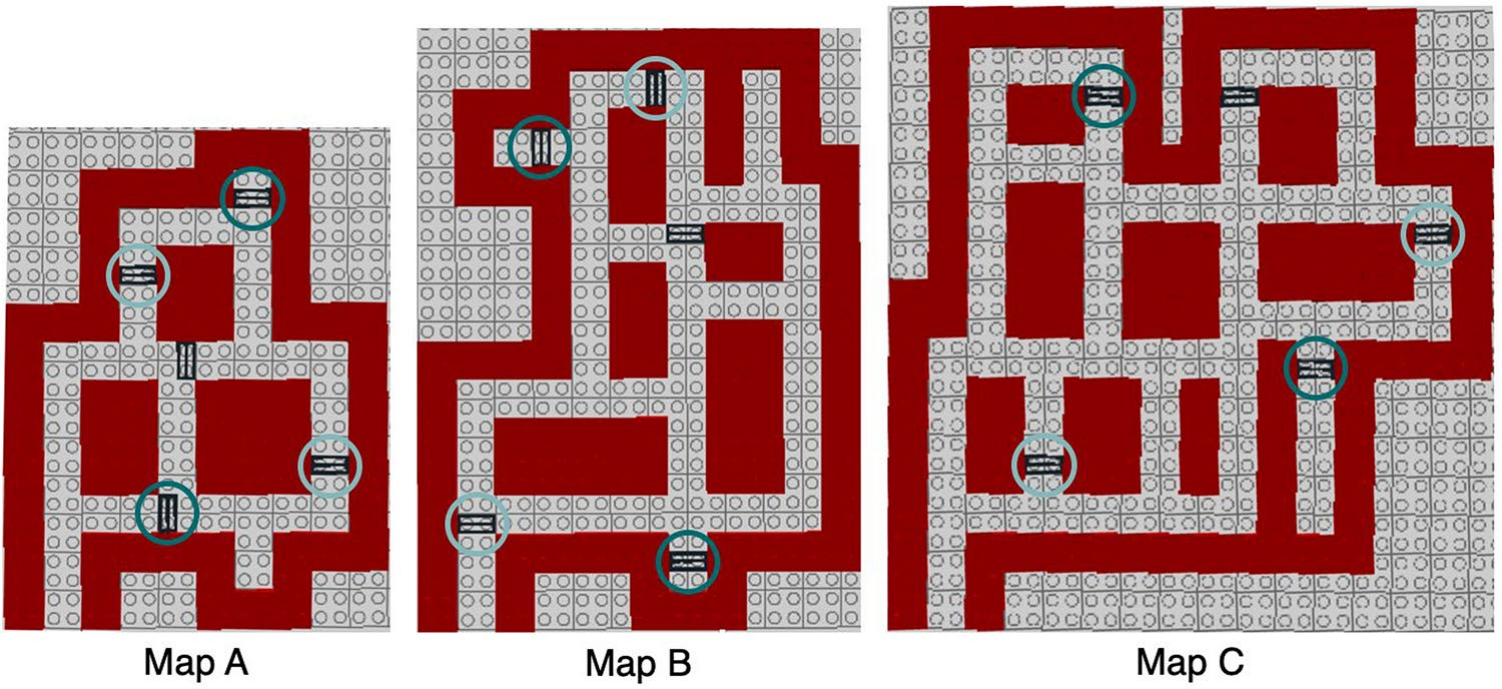
Routes for the navigation task. Subjects had to navigate the shortest route between the items that were associated to the predetermined locations with light circles, and between the ones with dark circles.

Furthermore, the software provides a distance accuracy score for each map. It thereby calculates the distance ratio for the distance between each landmark pair, for the reference map and the rebuilt maps. The ratio is the Euclidean distance between a landmark pair divided by the largest between-landmark Euclidean distance. Then, the mean distance error across landmark pairs on the rebuilt maps are computed. This score is subtracted from 1, yielding the distance accuracy score for each map^55^. An example of this analysis is given in Appendix A (Figure A.2, Supplementary Table 1).

The analysis was performed by two independent researchers, since the scores depend on placement of the landmarks by the researchers. This is not completely objective, since the landmarks are placed on intersections that the researcher thinks are the most similar. This is especially subjective when a map is (partly) not correct. If their scores deviated less than 0.05 from each other, the average was taken. When there was a larger deviation, consensus was reached on how to place the landmarks to calculate scores.

### Item placement task

#### Task

In the item placement task, participants had to indicate their remembered locations of the items. They received the original map but without location markings and items. They had to place the location markings on the spot they remembered, and place the corresponding items next to them. Onced placed, the items and location markings remained on the map, and the order of items was randomized. Both groups performed this task in their respective visual conditions.

#### Analysis

For each placed item, we calculated the error as the Euclidean distance to the correct location markings. The errors were divided by the maximum error possible, which was different for each location, to correct for this maximum error possible and for differences in map size. Therefore, the error score can be seen as distance of the placed item location from the correct location as a proportion of the largest Euclidean distance from the item location on the map. The mean error across locations was then computed.

### Route navigation task

#### Task

During the route navigation task, participants had to navigate two routes between two predetermined item locations (Fig. 2). They received the original map, including correct location markings, but without items. The participants were asked to navigate between two diferent item pairs using their right index finger, thereby taking the shortest path. At the start of both routes, the experimenter placed the right index finger of the participant on the first item location. The two routes were chosen such that they both are about the longest route on the map (Fig. 2). The direction of the routes was counterbalanced across participants.

Results from this task about the VH group will not be informative, as they can see the map an visually plan the shortest route. It will be more interesting to investigate whether the H group has a mental representation of the whole map that is comprehensive enough to plan and execute the shortest route between two items.

#### Analysis

For the two navigated routes, we computed the distance deviation from the shortest possible route. This deviation was corrected for the length of the shortest possible route. The mean of the two routes was taken to get a deviation score for each participant.

### Statistical analysis

All analyses were performed in MATLAB (MATLAB and Statistics Toolbox Release 2017b, The MathWorks, Inc., Natick, Massachusetts, United States). All tables with statistical results can be found in the supplemental material (Appendix B). To compare the outcomes of all tasks between maps, we used Wilcoxon signed-rank tests for paired samples, since the data was not normally distributed. To compare results of all tasks between the H and VH groups, we applied Wilcoxon rank sum tests for independent samples. Per task, we adjusted the *p*-values using the Bonferroni-Holm method for multiple comparisons correction. Additionally, we computed a Bayes factor (BF_01_) for each statistical comparison, to describe the likelihood of our results, given the relatively small sample size. Bayesian methods can analytically establish, given the data, whether the null hypothesis is more likely than the alternative hypothesis and vice versa^26,56,57^, and have been used in similar research^26^. Bayes factors indicate the likelihood of the null hypothesis over the alternative hypothesis. The smaller the BF_01_ (< 0.33), the stronger the suggestion that the alternative is more likely than the null hypothesis, thus strengthening significant differences that we found. Computations were performed in JASP (JASP Team, 2020. JASP, Version 0.13.1 [Computer software]). Because of non-normality of our data, we applied non-parametric Bayes factor calculation.

## Results

In short, the participants in this study learned three tactile maps of different complexities, including five item locations, by free exploration using their right index finger. We assessed cognitive map formation using four additional tasks. Participants first estimated relative distances between each item pair, which we analyzed by correlating the estimated with the correct distances, as well as by calculating over- and underestimation. Next, participants rebuilt the paths of the map using LEGO bricks, of which we analyzed correct relative placement of each node and intersection. Then, participants navigated the shortest route between two item pairs, of which we computed the deviation from the shortest route. Finally, participants placed the item locations back on the original map without item locations. Here, we calculated the distance between the indicated and the correct location. The results of these tasks are described below.

### Distance estimation performance

**Figure 3:**
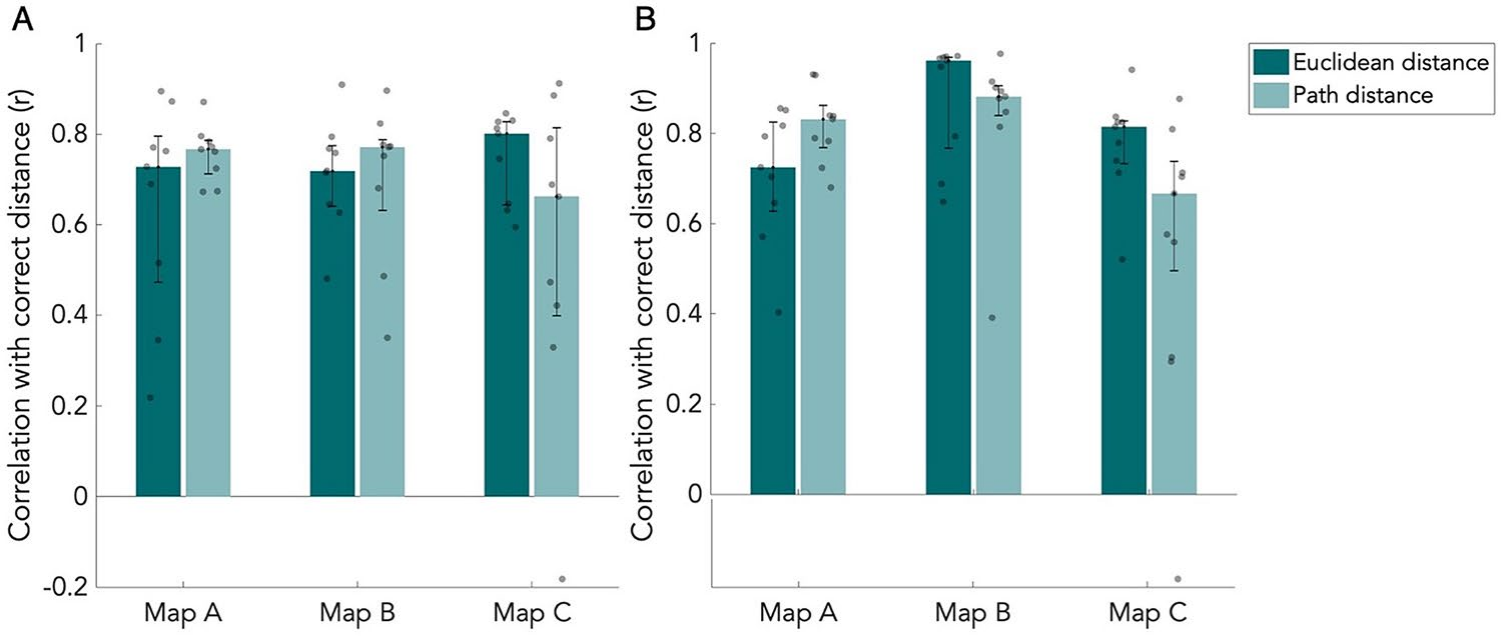
Correlations of the distance estimation task. Median correlation coefficient r of estimated distances with correct distances from the distance estimation task. Results are shown for both Euclidean and path distance for all three maps. Error bars indicate the 25th and 75th percentiles. Grey dots are individual data points. (**A**) Results of the haptic group (n = 9). (**B**) Results of the visuo-haptic group (n = 9).

Participants estimated relative Euclidean and path distances between each item pair. We analyzed their estimations using a correlation and an error analysis. The median correlation coefficients r from both the H and the VH groups are shown in Fig. 3. All correlations were were high and significantly different from zero, indicating that all participants had a good feeling of distances between items. Furthermore, from the results of the item placement task, we can infer that the participants remembered the item locations well. After correction for multiple comparisons, we found no significant differences between maps, distance types, or groups (Table 1). One thing to note here is that the Euclidean and path distances highly correlate on all maps (Map A: r = 0.97; Map B: r = 0.95; Map C: r = 0.88). The median performance is higher for the VH group than the H group on map B. This, however, is not significant after correction for multiple comparisons, and the effect is not present for map A and C (Table 1). Interestingly, the H group thus performed similarly as the VH group on this task, suggesting an accurate representation of distances in both groups. Bayes factors confirm that the evidence for differences in performance across maps or groups is very weak (Table 1).

**Figure 4:**
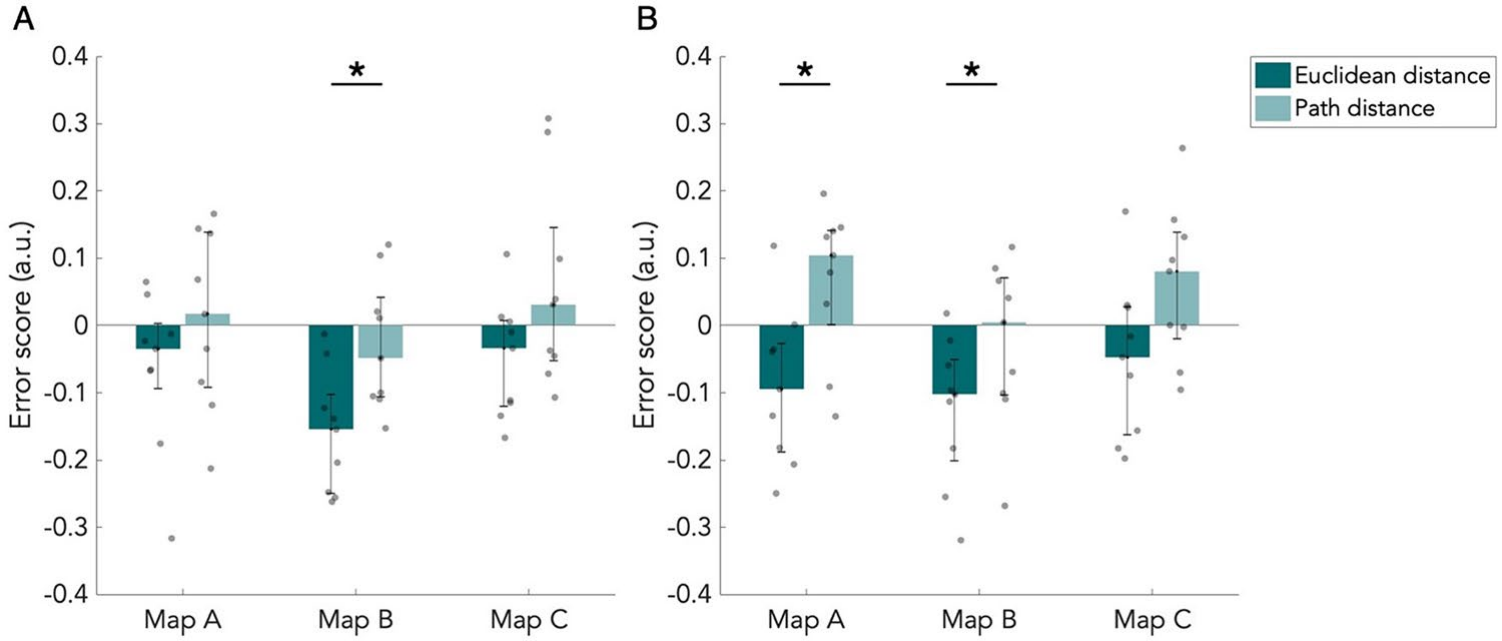
Error scores of the distance estimation task. Median error score of estimated distances, corrected for the maximum error possible. Results are shown for both Euclidean and path distance for all three maps. Error bars indicate the 25th and 75th percentiles. Grey dots are individual data points. (**A**) Results of the haptic group (n = 9). (**B**) Results of the visuo-haptic group (n = 9). *p < 0.05, corrected for multiple comparisons.

The median error scores for both groups are shown in Fig. 4. Overall, the errors were small: most estimations had a proportional deviation from the correct distance below 0.15. After multiple comparisons correction, we found a significant difference between the Euclidean and path distances types on map B in the H group (*p* < 0.05, W = − 45, BF_01_ = 0.034) and in the VH group (*p* < 0.05, W = − 45, BF_01_ = 0.048). On map B, path distances were estimated more accurately than Euclidean distances. Furthermore, we found a significant difference in the VH group on map A, between the distance types (*p* < 0.05, W = − 45, BF_01_ = 0.070), which was driven by an underestimation of Euclidean distances and overestimation of path distances. For both groups, the mean error of estimated path distances on map B was lower (Fig. 4). We did not find any significant difference between maps or groups after correction for multiple comparisons (Table 2), suggesting that the lack of difference in performance between the groups is also reflected in the results from this analysis. This lack of significance is reflected by Bayes factors (Table 2).

### Rebuilding performance

**Figure 5:**
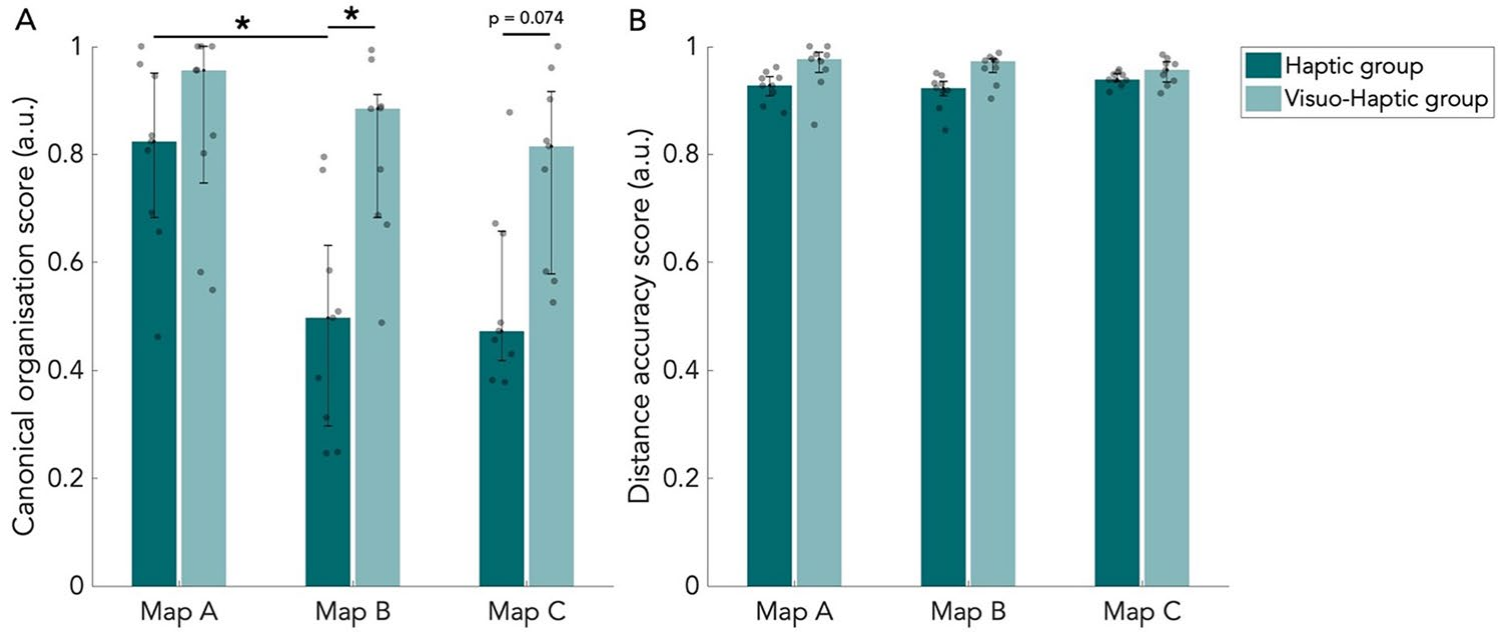
Scores of the rebuilding task. Median scores of the rebuilt maps. Results are shown for all three maps and both the haptic (n = 9) and visuo-haptic group (n = 9). Error bars indicate the 25th and 75th percentiles. Grey dots are individual data points. (**A**) Median canonical organization scores. (**B**) Median distance accuracy scores. *p < 0.05, corrected for multiple comparisons.

During the rebuilding task, participants rebuilt the paths of each map using LEGO bricks. All rebuilt maps can be found in the appendix (Figure A.3). We calculated canonical organization scores and distance accuracy scores for the rebuilt maps from the rebuilding task using GMDA software (Fig. 5). In the H group, the canonical organization score is high for map A, but for most participants below 0.5 for map B and C (Fig. 5A). The difference between map A and B was found to be significant (*p* < 0.05, W = 45, BF_01_ = 0.017; corrected for multiple comparisons). For the VH group, there was no difference in performance between the maps (Fig. 5A; Table 3).

We found a significant difference between the H and VH group on map B (*p* < 0.05, W = 55, BF_01_ = 0.269), and approaching significance between the groups on map C (*p* = 0.074, W = 57, BF_01_ = 0.350). Considering the rather small sample size, the difference between groups on map C probably would become significant if more participants are included. It thus seems that there is a higher performance by the VH group on the rebuilding task, but not on the least complex map (Table 3).

Concerning the distance accuracy of the rebuilt maps, we did not find significant differences between maps or groups after correcting for multiple comparisons (Fig. 5B; Table 4). This might indicate that the accurate representation of distances suggested from the distance estimation task is also reflected in the results from the rebuilding task. Bayes factors additionally do not reflect evidence for differences between maps or groups (Table 4).

Calculating the canonical organisation and distance accuracy scores was done by two independent researchers, since these scores highly depend on placement of the nodes by the researchers, which is not completely objective. The researchers reached agreement (difference in score < 0.05) for 61% of the maps. For the maps where the researchers had to reach consensus, there was a difference > 0.05 in the canonical organisation score. For these maps, the mean difference of the scores between the two researchers was 0.081.

### Item placement performance

**Figure 6:**
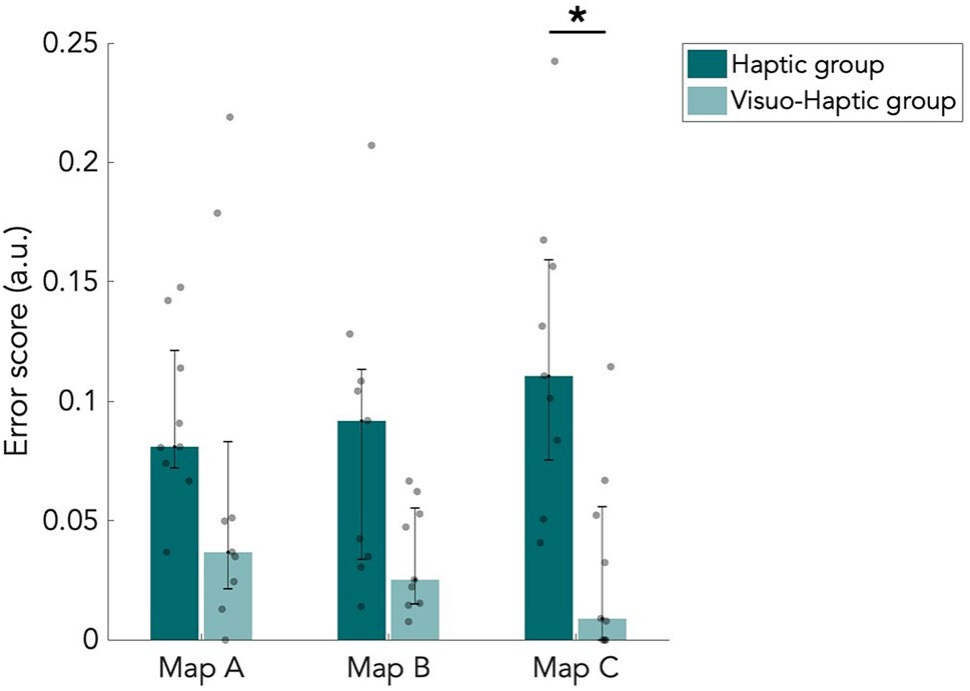
Error scores of the item placement task. Median error score of placed items, corrected for the maximum error possible. Results are shown for all three maps and both the haptic (n = 9) and visuo-haptic group (n = 9). Error bars indicate the 25th and 75th percentiles. Grey dots are individual data points. *p < 0.05, corrected for multiple comparisons.

Participants placed the items back on the maps during the item placement task. For each participant, we computed an error score as the mean Euclidean distance between the placed item and the correct location, corrected for the maximum error possible. Median error scores from both the H and VH groups are shown in Fig. 6. All participants performed well, since most participants had an error score below 0.12. This shows that most participants remembered where the items were located on the maps. The median error score was higher for the H group than the VH group. After correction for multiple comparisons, this was found to be significant on map C (*p* < 0.05, W = 54, BF_01_ = 0.260). Interestingly, the VH group thus did perform better than the H group, but only significantly on the most complex map (Table 5). However, considering the small sample size, performance on map A and B might become significantly different between the groups when more participants are included. We did not find a significant difference between maps within the groups, so map complexity did not have an effect on performance on the item placement task. Lack of significance is reflected by Bayes factors (Table 5).

### Route navigation performance

**Figure 7:**
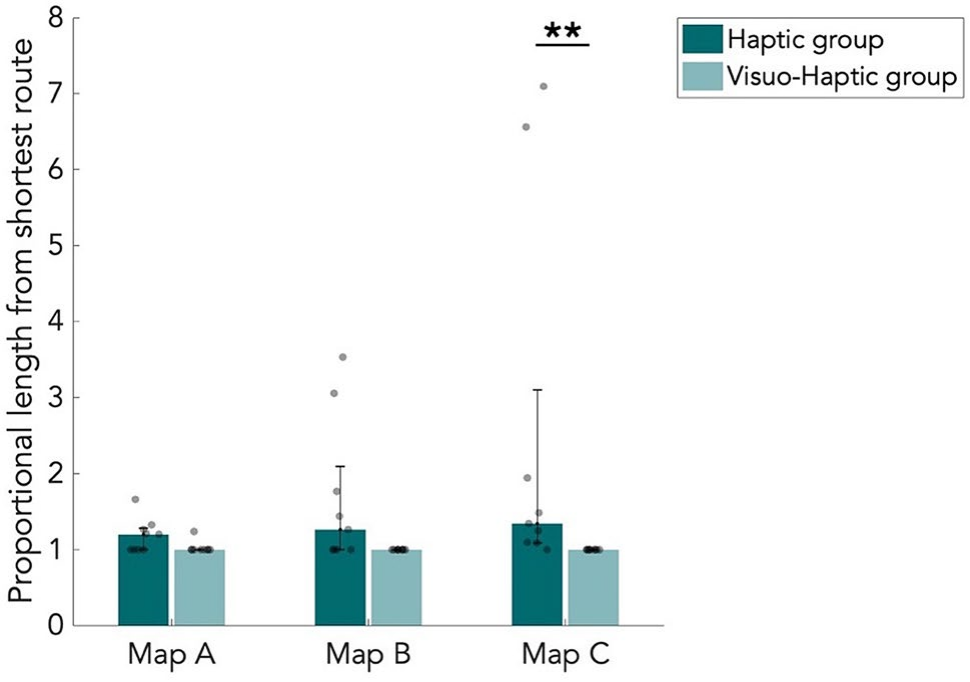
Median length of the navigated route during the navigation task, proportional to the shortest route of the navigation task. For each participant, the mean of the two navigated routes was taken first. Results are shown for all three maps and both the haptic (n = 9) and visuo-haptic group (n = 9). Error bars indicate the 25th and 75th percentiles. Grey dots are individual data points. **p < 0.01, corrected for multiple comparisons.

Of the two navigated routes during the route navigation task, we computed the average distance deviation from the shortest route as a proportion of the shortest route distance. The results of both the H and VH group are shown in Fig. 7. Eight out of nine VH participants had no deviation, while H participants did not navigate the shortest route more often (Fig. 7).

We found a significant difference between the H and VH group on map C (*p* < 0.01, W = 49.5). We did not find significant differences between maps of different complexity (Table 6). As in the item placement task, the VH group performed significantly better than the H group but only on the most complex map.

Across all tasks, the significant results that we find are confirmed by a low Bayes factor (BF_01_; Table 1–6). Such evidence for the alternative hypothesis is not shown by the Bayes factor for all other comparisons.

### Relation between task performance and end interviews

At the end of the experiment, participants were asked about their experience during the experiment and about navigation in daily life. We found no relationship between the reported learning strategy during the task or the daily life navigation strategy and the results of any of the tasks. 16 out of 18 participants reported that the tasks became (somewhat) easier with the second and third map, due to familiarization with the tasks. However, this was not reflected in the results and therefore does not explain the effects.

## Discussion

In the current study, we investigated cognitive map formation of an environment that is presented as a tactile map during a relatively short learning period, and how this compares to the acquisition of such a cognitive map via a combination of haptic and visual information. We used a combination of behavioral tasks, which allows for assessment of different kinds of spatial information, and egocentric as well as allocentric perspectives^1^. After learning three tactile maps of differing complexity, and five item locations on each map, participants performed four tasks to assess the accuracy of the formed cognitive maps. Overall, all participants, from both the haptic and the visuo-haptic group, performed well on all tasks for the least complex map, suggesting that they formed accurate cognitive maps. The VH group performed slightly better, but only on the more complex maps. The groups performed similarly on the distance estimation task. This result is in line with ideas of modality-independent coding of space in the brain. It furthermore indicates that people are able to form accurate cognitive maps of a tactile environment, with and without the use of vision and after a short learning period.

### Similar and accurate cognitive maps of the least complex map based on visual and haptic information

Overall, the participants of the haptic and the visuo-haptic group perform similar on all spatial tasks for the least complex map. This indicates they form similar and quite accurate cognitive maps of this tactile map. It also implies that there is not necessarily an advantage of visual input over only haptic input during navigation when having to infer spatial information from a cognitive map. This was suggested in previous research^52^, but with directions. There, people who learned routes haptically were able to infer directional information from their cognitive maps as good as people who learned routes visually. Besides, another study shows that drawings of maps improved substantially after blind participants had felt a tactile map compared to when they only had experienced the environment by walking^21^. It should be considered, however, that the scale of a tactile map is close to a map drawing, while the environment is experienced on a much larger scale when walking. These results might therefore say more about effectiveness of the scale than of the tactile/haptic modality.

Our findings that vision does not seem to have an advantage on the least complex map, are in line with ideas of modality-independent representations of space. This has been suggested in multiple lines of research^44–51^. These studies imply the integration of multiple input modalities into one amodal spatial representation, rather than the formation of a separate representation for each modality. For example, mental spatial representations have been found to be nearly independent of modality, and neural patterns of spatial representations from the haptic and visual modalities are highly similar^49,52^. Furthermore, the same brain regions are activated during spatial tasks involving different modalities or types of spatial information^45,51^. In our study, we don’t have a cross-modal condition to substantiate these findings, therefore, more empirical evidence is necessary to support these lines of work. We think, however, that especially our results of the distance estimation task might contribute to these ideas. Here, we found no differences in performance between groups, maps, or distance types. We argue that especially the high performance on Euclidean distance estimation is an indication of cognitive map formation. This metric was not presented explicitly on the maps, and suggest an overview-like representation. Furthermore, this measure considers a representation that supports an allocentric perspective, instead of only an egocentric perspective^1,3^. Important to note here, however, is that Euclidean and path distances are correlated, and allocentric and egocentric perspectives are not systematically disentangled. Therefore, we must be careful with hard claims about this. Furthermore, participants were instructed to find the shortest routes between locations, but these were not given. It could thus be that some participants considered slightly different routes as the shortest. These routes, however, probably did not differ much in length, so we suspect that this did not affect the path distance estimations substantially. Nonetheless, this is a point to be considered in the experimental design of further studies. When considering the error analysis of the distance estimation task, we reveal a tendency to underestimate Euclidean, and overestimate path distance. A tendency to underestimate Euclidean distance has been found after learning a map and after navigating a real world environment^58^. The opposite, however, was found in a study on distance estimation after route navigation in a virtual environment^59^. Nonetheless, it is difficult to compare these findings to our results, since participants in these studies used vision, and they navigated a large-scale environment where the perspective during navigation is different. The tendencies of underestimation of Euclidean distance and overestimation of path distance in our study might have been influenced by the scale the participants had to use for the estimations. They had to transform the largest Euclidean and path distance possible on the map to the size of the ruler, and then scale the item-pair distances to this. The length of the ruler was considerably similar to the maximum Euclidean distance, while the maximum path distance was larger, especially on the larger maps.

On the more complex maps B and C, our results seem to point towards an advantage of vision over haptic information. On the rebuilding task, the canonical organisation score decreases with complexity. When thinking in terms of how useful the rebuilt map would be to use as a reference during navigation, the rebuilt maps B and C of the H group would probably be not very effective (see Figure A.3). The VH group rebuilt maps that would be quite useful during navigation. The distance scores were overall very high. This is not very surprising, however, since the absolute size of the maps is not large, and participants were restricted during building by the size of the building plate. Furthermore, the learning and rebuilding tasks took place in peripersonal space. Therefore, participants could get a good idea of distances between nodes and the size of the map using the position of the finger compared to the body position. Another thing to note here, is that the scoring of the rebuilt maps is not completely objective, and depends on placement of the nodes by the two researchers. They reached agreement for 61% of the maps, and for the maps where they did not reach agreement, their scores differed only with a mean of 0.081. There is, however, to our knowledge no method to objectively analyze a rebuilt map and all its attributes. Besides, because of the rectangular structures on the map, it could be that on the more complex maps, subjects did not have good memory of the map, but only of some intersections and rectangular formations. They could have ordered these in a sensible way and spontaneously resembled the original map, as building a map by humans followes certain principles.

On the item placement task, the advantage of vision becomes especially clear on the most complex map. Decreased performance with increasing complexity is in line with earlier results^36^. Here, participants made more errors recalling object position when there were more objects present, and thus complexity increased. The same pattern holds for the navigation task. The haptic group shows a larger spread in performance on the more complex maps. It is not surprising, however, that all participants in the VH group took the shortest routes, since they could see the map and the locations, and thus visually plan the route. This task is therefore less suitable to give suggestions about the advantage of vision on cognitive map accuracy. However, it still indicates that many participants know the maps well enough to navigate the routes.

### Relevance and future directions

In accordance with previous research on tactile map exploration, our results indicate that people can form cognitive maps of tactile models of city-like environments. We thereby provide a comprehensive view by combining several spatial aspects of a cognitive map, instead of studying only one aspect such as recall of routes^23,37^, reproduction of map layouts^21,26^, or estimation of distances between learned locations^39^.

Our findings in this experiment might have implications for persons who are not able to use visual information in their daily lives, for example persons with a visual impairment (PVIs). Although this study is not performed with participants who are visually impaired, we still think that the results are promising. We show that presenting a fairly simple environment haptically allows for the formation of a cognitive map that is comparable to a cognitive map formed using visual and haptic information combined. Such a mental representation of the map could support navigation in the real environment, and our results are in line with research that shows the effectiveness of tactile maps to support blind people in wayfinding and orientation^20,23,25–30,36,37^. To construct a tactile map, one should take the complexity into account, as well as how much and what kind of information is presented, since we do find some differences between map A, B and C. To be able to give indications about cognitive maps and navigation based on haptic information in persons who are actually visually impaired, a logical next step in this research would be to perform a similar experiment with visually impaired participants. A follow-up experiment could for example be to investigate how the mental representation acquired through the tactile map would support navigation by visually impaired participants in the real-world environment. This would give an indication of whether exploring a tactile map first, and the cognitive map acquired through it, would be beneficial to actual navigation. When generalising experimental findings in sighted persons to PVIs, however one should always be careful. Many studies have shown that there are differences in performance between vision-restricted or blindfolded sighted persons and PVIs. Besides, there could be differences between PVIs, due to for example specific impairments, the age when they acquired the impairment, and how much visual experience they have. Furthermore, differences in the use of egocentric and/or allocentric perspectives should be taken into account.

Furthermore, to investigate whether a similar cognitive map will form in real-world navigation, one should consider differences between small-scale and large-scale environments. There is one study where participants perform better on small-scale haptic spatial tasks than on their large-scale version^60^. Tactile maps are less noisy than the real-world environment, contain less irrelevant information, and can be explored much faster^61^. An important thing to consider when comparing small-scale with large-scale environments, is the use of allocentric and egocentric perspectives. From a navigation perspective, when navigating in a real world, one experienced this mostly egocentrically. A small-scale tactile map is mostly perceived allocentrically (from above), which may have an impact on the spatial representation one builds up. Follow-up studies that adress cognitive maps based on haptic information in large-scale environments are therefore necessary to draw firmer conclusions about real-life navigation without vision.

One limitation of the bird view-like presentation of the tactile map, is that participants could have reproduced locations and distances by representing them relative to the body instead of actually learning them via navigation. This makes it difficult to give more explicit indications about for example disentangling allocentric and egocentric perspectives. Furthermore, in a future experiment, it would be better to have the sighted participants only see the maps during the map learning, but not during the other tasks. This would isolate the effect of vision during the formation of the cognitive map. In the current experiment, vision could have had an influence on the task performance itself as well, instead of only on cognitive map formation. Another limitation is that in the visuo-haptic condition, there is not an exact way of knowing how much visual and how much haptic information contributed to the performance of the VH group. However, since vision is the dominant sense, it is almost certain that participants made use of visual input. Additionally, they were instructed to explore the maps haptically and use this sensory input. Therefore, we think that the VH group used both visual as well as haptic information during the experiment.

## Conclusion

The results from this experiment suggest that all participants, using only haptic or a combination of haptic and visual information, could form accurate cognitive maps of the least complex environment, even in a relatively short learning period. These cognitive maps contained various types of spatial information, and they might support egocentric as well as allocentric perspectives. This results is in line with ideas of modality-independent representation of space, however, more empirical evidence is necessary to support this claim. With increasing map complexity, vision seemed to have an advantage over haptic information on most spatial tasks. Except for the distance estimation task, suggesting that participants still formed a representations of the more complex maps.

## Supplementary Information

Supplementary Information.

## Data Availability

The data and all materials for the experiments reported here are available.
