# Supplementary material for: Cognitive map formation through haptic and visual exploration of tactile city-like maps
Source: Sci Rep. 2021 Jul 27;11:15254. doi: 10.1038/s41598-021-94778-1 (PMC8316501; doi:10.1038/s41598-021-94778-1)
Supplement: Supplementary file 1 — Supplementary Information. [file 41598_2021_94778_MOESM1_ESM.docx]

**Appendix A. Map rebuilding task**


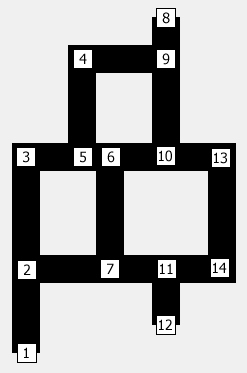


**Figure A.1. Example of landmark placement in GMDA software**. The numbered landmarks as they were placed in the GMDA software on original map A. These landmarks were also placed on each rebuild map, after which the GMDA compared the landmark placing on the original and rebuilt maps, and calculated various scores for the rebuilt maps. (Gardony map drawing analyzer: Software for quantitative analysis of sketch maps, version 1, https://www.aarongardony.com/tools/map-drawing-analyzer^55^)


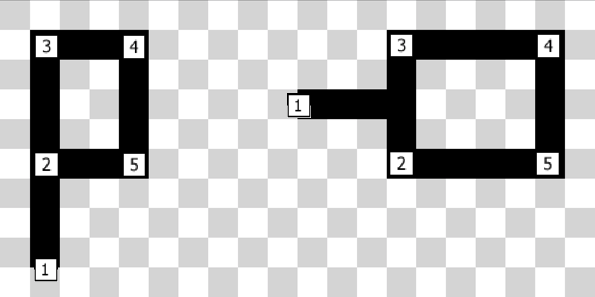


**Figure A.2. Landmark placement in GMDA on an example reference map (left) and an example rebuilt map (right).** Paths are draws as black lines, where the width corresponds to the width of the path in the LEGO maps. (Gardony map drawing analyzer: Software for quantitative analysis of sketch maps, version 1, https://www.aarongardony.com/tools/map-drawing-analyzer^55^)

**Example of canonical organisation and distance score calculation**

For the canonical organisation score, the GMDA software first determines the North-South and East-West relations between all landmark pairs on the reference map (left, Figure A.2), and on the rebuilt map (right, Figure A.2; Table 1). The software then determines whether the N-S and E-W relations on the rebuilt map are correct for each landmark pair. For example, landmark 2 is located North of 1 on the reference map, but Southeast on the rebuilt map. Both N-S and E-W relations are not correct, yielding a score of 0 for this pair. Landmark 3 is located North of 1 on the reference map, but Northeast on the rebuilt map. The N-S relation is correct, but the E-W relation not, giving a score of 0.5 for this pair. This calculation is performed for each landmark pair. The mean across pairs is the resulting canonical organisation score (Table 1).

Furthermore, the software provides a distance accuracy score for each map. It thereby calculates the distance ratio for the distance between each landmark pair, for the reference map and the rebuilt maps (Table 1). The ratio is the Euclidean distance between a landmark pair divided by the largest between-landmark Euclidean distance. The largeste between-landmark distance is 7.62 for the example reference map, and 8.25 for the example rebuilt map, both between landmark 1 and 4. Then, the mean distance ratio error across landmark pairs on the rebuilt maps are computed. This score is subtracted from 1, yielding the distance accuracy score of 0.88 (Table 1).

**Supplementary Table 1. Example CanOrg and DistAcc score calculation by GMDA software.** (GMDA; Software for quantitative analysis of sketch maps, version 1, https://www.aarongardony.com/tools/map-drawing-analyzer)^55^

| **Landmark pair** | **N-S and E-W relations reference map** | **N-S and E-W relations rebuilt map** | **CanOrg score** | **Distance ratio reference map** | **Distance ratio rebuilt map** | **DistAcc score** |
| --- | --- | --- | --- | --- | --- | --- |
| 1 – 2 | N/- | S/E | 0 | 3/7.62 = 0.39 | 3.61/8.25 = 0.44 | 0.05 |
| 1 – 3 | N/- | N/E | 0.5 | 7/7.62 = 0.92 | 3.61/8.25 = 0.44 | 0.48 |
| 1 – 4 | N/E | N/E | 1 | 7.62/7.62 = 1 | 8.25/8.25 = 1 | 0 |
| 1 – 5 | N/E | S/E | 0.5 | 4.24/7.62 = 0.56 | 8.25/8.25 = 1 | 0.44 |
| 2 – 3 | N/- | N/- | 1 | 4/7.62 = 0.52 | 4/8.25 = 0.48 | 0.04 |
| 2 – 4 | N/E | N/E | 1 | 5/7.62 = 0.66 | 5/8.25 = 0.61 | 0.05 |
| 2 – 5 | -/E | -/E | 1 | 3/7.62 = 0.39 | 3/8.25 = 0.36 | 0.03 |
| 3 – 4 | -/E | -/E | 1 | 3/7.62 = 0.39 | 3/8.25 = 0.36 | 0.03 |
| 3 – 5 | S/E | S/E | 1 | 5/7.62 = 0.66 | 5/8.25 = 0.61 | 0.05 |
| 4 – 5 | S/- | S/- | 1 | 4/7.62 = 0.52 | 4/8.25 = 0.48 | 0.04 |
| Mean across pairs | | | **0.8** | Mean across pairs, subtracted from 1 | | **0.88** |


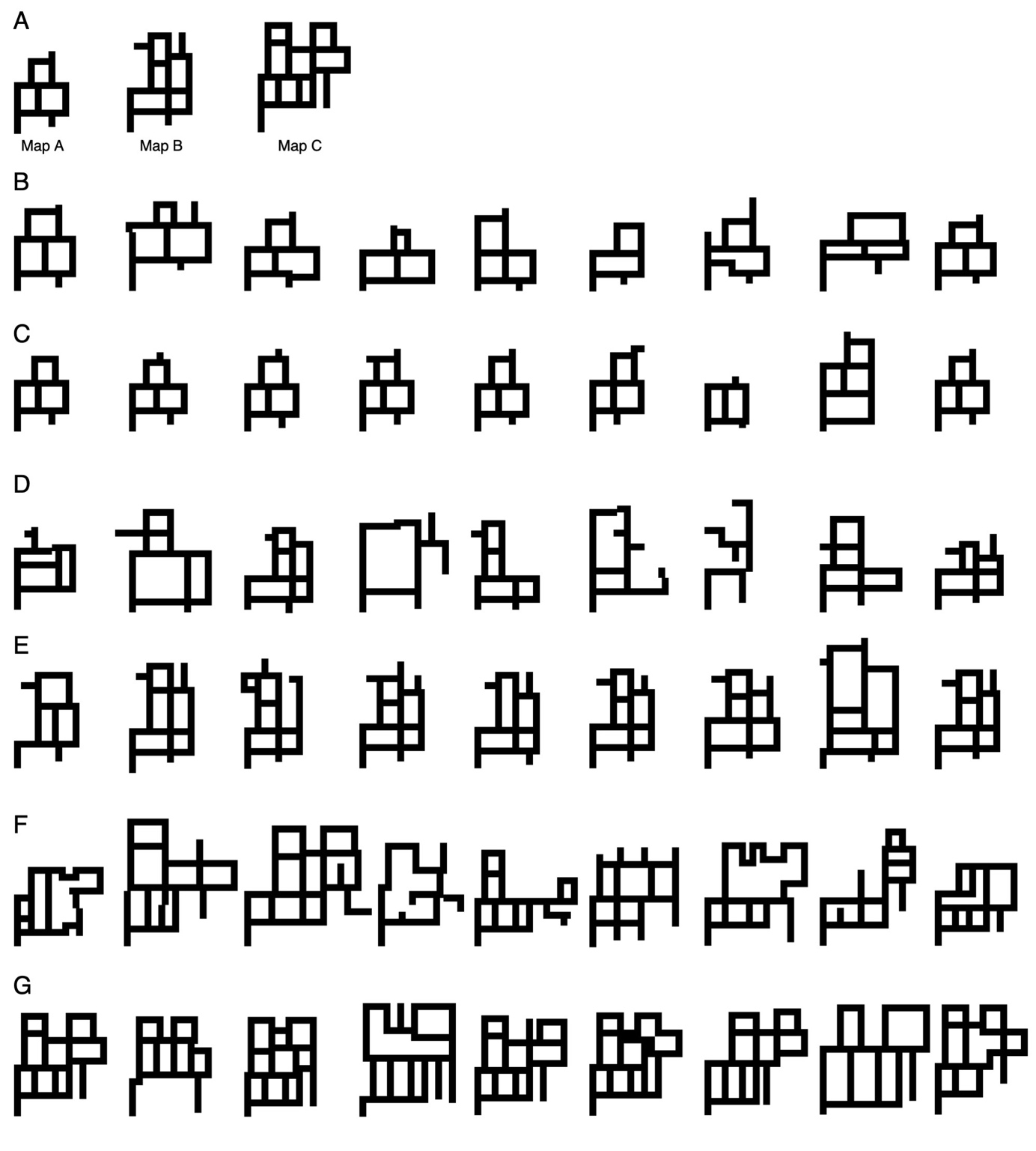


**Figure A.3**. **Individual rebuilt maps.** Standardized images of the individual rebuilt map paths by all subjects from the rebuilding task. **A**. Correct maps. **B**. Map A by the haptic group. **C**. Map A by the visuo-haptic group. **D**. Map B by the haptic group. **E**. Map B by the visuo-haptic group. **F**. Map C by the haptic group. **G**. Map C by the visuo-haptic group.

**Appendix B. Statistical results**

**Table 1.** **Statistical results of the correlation analysis of the distance estimation task.** Results of the statistical Wilcoxon signed-rank (within groups) and Wilcoxon rank sum (between groups) tests of the correlation analysis of the distance estimation task. Shown are uncorrected p-values, number of comparisons, α-values after Bonferroni-Holm multiple comparisons correction, p-values after Bonferroni-Holm multiple comparisons correction, test statistic W, and Bayes factors (BF_01_).

| **Test**  **(group/map/distance type)** | | **p**  **(uncorrected)** | **n comparisons** | **α**  **(corrected)** | **p**  **(corrected)** | **W** | **BF_01_** |
| --- | --- | --- | --- | --- | --- | --- | --- |
| Haptic | A-B Eucl | 0.426 | 9 | 0.01 | 1 | -11 | 1.470 |
|  | A-C Eucl | 0.426 | 9 | 0.01 | 1 | -15 | 1.539 |
|  | B-C Eucl | 0.652 | 9 | 0.0167 | 1 | -15 | 1.760 |
|  | A-B path | 0.734 | 9 | 0.0167 | 1 | 5 | 1.757 |
|  | A-C path | 0.129 | 9 | 0.0063 | 1 | 9 | 0.968 |
|  | B-C path | 0.301 | 9 | 0.0083 | 1 | 0 | 1.375 |
|  | A Eucl-path | 0.250 | 9 | 0.0071 | 1 | -29 | 1.283 |
|  | B Eucl-path | 1 | 9 | 0.025 | 1 | 1 | 1.919 |
|  | C Eucl-path | 0.098 | 9 | 0.0056 | 0.879 | 11 | 0.891 |
| Visuo-haptic | A-B Eucl | 0.039 | 9 | 0.0056 | 0.352 | -29 | 0.531 |
|  | A-C Eucl | 0.203 | 9 | 0.0125 | 0.813 | -13 | 1.233 |
|  | B-C Eucl | 0.203 | 9 | 0.0125 | 0.813 | 15 | 1.202 |
|  | A-B path | 0.496 | 9 | 0.025 | 0.992 | -15 | 1.704 |
|  | A-C path | 0.055 | 9 | 0.0083 | 0.328 | 35 | 0.759 |
|  | B-C path | 0.039 | 9 | 0.0056 | 0.352 | 0 | 0.619 |
|  | A Eucl-path | 0.039 | 9 | 0.0063 | 0.313 | -41 | 0.502 |
|  | B Eucl-path | 0.652 | 9 | 0.025 | 0.992 | 1 | 1.755 |
|  | C Eucl-path | 0.074 | 9 | 0.01 | 0.371 | 27 | 0.834 |
| Between  groups | A Eucl | 0.796 | 6 | 0.0167 | 1 | 82 | 2.326 |
|  | A path | 0.113 | 6 | 0.0125 | 0.454 | 67 | 1.036 |
|  | B Eucl | 0.019 | 6 | 0.0083 | 0.113 | 59 | 0.426 |
|  | B path | 0.019 | 6 | 0.0083 | 0.113 | 59 | 0.522 |
|  | C Eucl | 0.730 | 6 | 0.0167 | 1 | 81 | 2.349 |
|  | C path | 1 | 6 | 0.025 | 1 | 85 | 2.434 |

**Table 2.** **Statistical results of the error analysis of the distance estimation task.** Results of the statistical Wilcoxon signed-rank (within groups) and Wilcoxon rank sum (between groups) tests of the error analysis of the distance estimation task. Shown are uncorrected p-values, number of comparisons, α-values after Bonferroni-Holm multiple comparisons correction, p-values after Bonferroni-Holm multiple comparisons correction, test statistic W, and Bayes factors (BF_01_).

| **Test**  **(group/map/distance type)** | | **p**  **(uncorrected)** | **n comparisons** | **α**  **(corrected)** | **p**  **(corrected)** | **W** | **BF_01_** |
| --- | --- | --- | --- | --- | --- | --- | --- |
| Haptic | A-B Eucl | 0.055 | 9 | 0.0083 | 0.328 | 33 | 0.698 |
|  | A-C Eucl | 0.570 | 9 | 0.025 | 1 | -9 | 1.675 |
|  | B-C Eucl | 0.012 | 9 | 0.0063 | 0.094 | -33 | 0.247 |
|  | A-B path | 0.359 | 9 | 0.0167 | 1 | 19 | 1.463 |
|  | A-C path | 0.570 | 9 | 0.025 | 1 | -27 | 1.606 |
|  | B-C path | 0.203 | 9 | 0.0125 | 0.813 | 0 | 1.211 |
|  | A Eucl-path | 0.027 | 9 | 0.0071 | 0.191 | -35 | 0.448 |
|  | B Eucl-path | 0.004 | 9 | 0.0056 | 0.035 * | -45 | 0.034 |
|  | C Eucl-path | 0.074 | 9 | 0.01 | 0.371 | -39 | 0.766 |
| Visuo-haptic | A-B Eucl | 0.359 | 9 | 0.0167 | 1 | 9 | 1.369 |
|  | A-C Eucl | 0.359 | 9 | 0.0167 | 1 | -9 | 1.425 |
|  | B-C Eucl | 0.074 | 9 | 0.0125 | 0.297 | -25 | 0.839 |
|  | A-B path | 0.020 | 9 | 0.0071 | 0.137 | 41 | 0.368 |
|  | A-C path | 1 | 9 | 0.05 | 1 | -43 | 1.794 |
|  | B-C path | 0.039 | 9 | 0.01 | 0.195 | 0 | 0.528 |
|  | A Eucl-path | 0.004 | 9 | 0.0056 | 0.035 * | -45 | 0.070 |
|  | B Eucl-path | 0.004 | 9 | 0.0056 | 0.035 * | -45 | 0.048 |
|  | C Eucl-path | 0.020 | 9 | 0.0071 | 0.137 | -31 | 0.378 |
| Between  groups | A Eucl | 0.436 | 6 | 0.0083 | 1 | 76 | 2.120 |
|  | A path | 0.436 | 6 | 0.01 | 1 | 76 | 1.963 |
|  | B Eucl | 0.387 | 6 | 0.0083 | 1 | 75 | 2.013 |
|  | B path | 0.931 | 6 | 0.0167 | 1 | 84 | 2.379 |
|  | C Eucl | 0.931 | 6 | 0.025 | 1 | 84 | 2.339 |
|  | C path | 0.796 | 6 | 0.0167 | 1 | 82 | 2.383 |
| * p < 0.05 | | | | | | | |

**Table 3.** **Statistical results of the canonical organization analysis of the rebuilding task.** Results of the statistical Wilcoxon signed-rank (within groups) and Wilcoxon rank sum (between groups) tests of the canonical organization scores of the rebuilding task. Shown are uncorrected p-values, number of comparisons, α-values after Bonferroni-Holm multiple comparisons correction, p-values after Bonferroni-Holm multiple comparisons correction, test statistic W, and Bayes factors (BF_01_).

| **Test (group/map)** | | **p (uncorrected)** | **n comparisons** | **α (corrected)** | **p (corrected)** | **W** | **BF_01_** |
| --- | --- | --- | --- | --- | --- | --- | --- |
| Haptic | A-B | 0.004 | 9 | 0.0056 | 0.035 * | 45 | 0.017 |
|  | A-C | 0.020 | 9 | 0.0083 | 0.117 | 23 | 0.387 |
|  | B-C | 0.301 | 9 | 0.01 | 1.504 | -9 | 1.580 |
| Visuo-haptic | A-B | 0.570 | 9 | 0.025 | 0.992 | 3 | 1.662 |
|  | A-C | 0.496 | 9 | 0.0125 | 1 | 17 | 1.620 |
|  | B-C | 0.496 | 9 | 0.0167 | 1 | 27 | 1.682 |
| Between groups | A | 0.420 | 9 | 0.0125 | 1 | 76 | 1.904 |
|  | B | 0.006 | 9 | 0.0063 | 0.045 * | 55 | 0.269 |
|  | C | 0.011 | 9 | 0.0071 | 0.074 | 57 | 0.350 |
|  |  |  |  |  |  | * p < 0.05 | |

**Table 4.** **Statistical results of the distance accuracy analysis of the rebuilding task.** Results of the statistical Wilcoxon signed-rank (within groups) and Wilcoxon rank sum (between groups) tests of the distance accuracy scores of the rebuilding task. Shown are uncorrected p-values, number of comparisons, α-values after Bonferroni-Holm multiple comparisons correction, p-values after Bonferroni-Holm multiple comparisons correction, test statistic W, and Bayes factors (BF_01_).

| **Test (group/map)** | | **p (uncorrected)** | **n comparisons** | **α (corrected)** | **p (corrected)** | **W** | **BF_01_** |
| --- | --- | --- | --- | --- | --- | --- | --- |
| Haptic | A-B | 0.734 | 9 | 0.025 | 0.922 | -7 | 1.707 |
|  | A-C | 0.250 | 9 | 0.0083 | 1 | -20 | 1.299 |
|  | B-C | 0.039 | 9 | 0.0071 | 0.273 | -33 | 0.545 |
| Visuo-haptic | A-B | 0.461 | 9 | 0.0167 | 1 | 17 | 1.626 |
|  | A-C | 0.301 | 9 | 0.01 | 1 | 19 | 1.468 |
|  | B-C | 0.426 | 9 | 0.0167 | 1 | 27 | 1.599 |
| Between groups | A | 0.018 | 9 | 0.0063 | 0.140 | 59 | 0.608 |
|  | B | 0.008 | 9 | 0.0056 | 0.070 | 56 | 0.339 |
|  | C | 0.286 | 9 | 0.0083 | 1 | 73 | 1.790 |

**Table 5.** **Statistical results of the item placement task.** Results of the statistical Wilcoxon signed-rank (within groups) and Wilcoxon rank sum (between groups) tests of the item placement task. Shown are uncorrected p-values, number of comparisons, α-values after Bonferroni-Holm multiple comparisons correction, p-values after Bonferroni-Holm multiple comparisons correction, test statistic W, and Bayes factors (BF_01_).

| **Test (group/map)** | | **p (uncorrected)** | **n comparisons** | **α (corrected)** | **p (corrected)** | **W** | **BF_01_** |
| --- | --- | --- | --- | --- | --- | --- | --- |
| Haptic | A-B | 0.652 | 9 | 0.025 | 1 | 27 | 1.808 |
|  | A-C | 0.250 | 9 | 0.01 | 1 | -19 | 1.327 |
|  | B-C | 0.426 | 9 | 0.0167 | 1 | -25 | 1.633 |
| Visuo-haptic | A-B | 0.301 | 9 | 0.01 | 1 | 25 | 1.397 |
|  | A-C | 0.027 | 9 | 0.0063 | 0.219 | 19 | 0.545 |
|  | B-C | 0.652 | 9 | 0.025 | 1 | 7 | 1.735 |
| Between groups | A | 0.081 | 9 | 0.0071 | 0.539 | 65.5 | 1.191 |
|  | B | 0.077 | 9 | 0.0071 | 0.539 | 65 | 0.916 |
|  | C | 0.004 | 9 | 0.0056 | 0.036 * | 54 | 0.260 |
|  |  |  |  |  |  | * p < 0.05 | |

**Table 6.** **Statistical results of the navigation task.** Results of the statistical Wilcoxon signed-rank (within groups) and Wilcoxon rank sum (between groups) tests of the route navigation task. Shown are uncorrected p-values, number of comparisons, α-values after Bonferroni-Holm multiple comparisons correction, p-values after Bonferroni-Holm multiple comparisons correction, test statistic W, and Bayes factors (BF_01_).

| **Test (group/map)** | | **p (uncorrected)** | | **n comparisons** | **α (corrected)** | **p (corrected)** | **W** | **BF_01_** |
| --- | --- | --- | --- | --- | --- | --- | --- | --- |
| Haptic | A-B | 0.328 | | 9 | 0.0083 | 1 | 1 | 1.474 |
|  | A-C | 0.301 | | 9 | 0.0083 | 1 | -15 | 1.311 |
|  | B-C | 0.640 | | 9 | 0.0125 | 1 | 2 | 1.685 |
| Visuo-haptic | A-B | 1 | | 9 | 0.0167 | 1 | 4.5 | NaN^◊^ |
|  | A-C | 1 | | 9 | 0.0167 | 1 | 4.5 | NaN^◊^ |
|  | B-C | 1 | | 9 | 0.025 | 1 | 0 | NaN^◊^ |
| Between groups | A | 0.070 | | 9 | 0.0071 | 0.491 | 67 | 1.068 |
|  | B | 0.029 | | 9 | 0.0063 | 0.235 | 63 | NaN^◊^ |
|  | C | 0.0004 | | 9 | 0.0056 | 0.004 ** | 49.5 | NaN^◊^ |
|  |  |  | ** p < 0.01  ^◊^The variance in one or both samples is 0 | | | | | |
